# From Information to Satisfaction: Unravelling the Impact of Sustainability Label on Fish Liking Experiences

**DOI:** 10.3390/foods14050890

**Published:** 2025-03-05

**Authors:** Giovanni Fiorile, Sharon Puleo, Francesca Colonna, Teresa Del Giudice, Rossella Di Monaco

**Affiliations:** 1 Department of Agricultural Sciences, University of Naples Federico II, 80055 Portici, Italy; giovanni.fiorile@unina.it (G.F.); francesca.colonna@unina.it (F.C.); teresa.delgiudice@unina.it (T.D.G.); rossella.dimonaco@unina.it (R.D.M.); 2 Centre for Food Innovation and Development in the Food Industry, University of Naples Federico II, 80055 Portici, Italy

**Keywords:** sustainable consumption, consumers’ awareness, bluefin tuna, anchovies

## Abstract

Fish sustainability has become an ever more important issue in recent years, as increases in consumption are leading to overfishing practices, resulting in the depletion of the seas and environmental damage. Therefore, fish companies have been adhering to sustainability programs, although these sustainable practices are not well valued and thus well known by end consumers. Therefore, this study aimed to assess the impact of sustainability label information on the hedonic perception of a consumer group regarding two fish species threatened by overfishing: European anchovy (*Engraulis encrasicolus*) and Bluefin tuna (*Thunnus thynnus*). The approach used was a blind–expected–informed evaluation. The results showed a positive perception of the species with the sustainability label by recording higher informed hedonic scores than blind ones. Thus, in conclusion, fish sustainability positively influenced the consumers, increasing their liking scores from the blind to the informed session. This study can expand previous knowledge on environmental sustainability, especially fisheries sustainability, and understand the relationships between sustainability eco-labels and consumer behaviour.

## 1. Introduction

As the world’s population grows increasingly, the demands for food are growing, especially in developed countries. Meeting everyone’s demands implies an over-exploitation of resources, so the most effective way to limit damage to the environment is through sustainability [1]. The concept of food sustainability has declined in several versions, and it can be stated that consumers’ awareness of environmental issues is growing [2,3,4]. As such, food companies have changed their modalities of production, which now respond to higher levels of sustainability. Specifically, to effectively demonstrate and ensure compliance with the sustainability criteria, food companies use eco-labels. According to the OECD (1991), “eco-label” is intended for the seals of approval given to products that are proposed to have fewer impacts on the environment than similar products. This is why the aim of the eco-label is to create clear and transparent communication between the producing company and consumers or stakeholders [5]. Since the beginning of the century, eco-labels have become increasingly important and accompany more and more products year by year [5]. As reported by other authors [6], external information, especially sustainability, has a positive effect on the propensity to purchase and the satisfaction of consumers, who feel part of a positive process towards the environment. There are more than 400 eco-labels worldwide, and most of them cover several food products [7]. Focusing attention on food products, close to 50 different labels worldwide are associated with the sustainability of seafood have been found [8]. Actually, the increasing consumption of fish products over the years has led to the need for sustainability programmes to prevent damage to fish stocks and preserve the sea [3,9,10]. Fishing sustainability requires that fishing practices are carried out in compliance with rules concerning quantities fished, fishing or farming techniques, respect for fishing times (growth and reproduction) and avoidance of damage to the sea [9]. As reported by other authors, in 2018, 14% of wild-caught and farmed species were certified by the nine voluntary sustainability standards operating globally, and this value will increase over the years [5]. Of the various sustainability labels available in European markets, the Marine Stewardship Council (MSC, www.msc.org accessed on 20 February 2024) label is among the most common. MSC is an international certification organisation for sustainable fishing practices (covering more than 20,000 products, MSC Annual Report 2022–2023, https://www.msc.org/about-the-msc/reports-and-brochures accessed on 20 February 2024) mainly aimed at reducing overfishing, protecting species and managing fishing practices at sea in the fairest way possible [11]. Moreover, MSC is the best-known label and has greater credibility among companies and consumers [12]. The MSC sustainability certification is based on performance indices that assign points to the fishery. The highest score achievable is 100—a fishery must score at least 60 performance indices points to qualify for certification [12].

Many studies have been conducted on eco-labels' effect on food evaluation [4]. Carefully analysing the significant results reported in the literature, tendentially, there is a positive effect of eco-labels on the consumer preference to buy sustainable fish and a higher willingness to pay, especially when consumers are informed about overfishing and sea depletion issues [13,14,15]. Thus, if a consumer perceives wild-capture fisheries as harmful to fish stocks and prefers farmed fish, the application of a sustainability label on wild-caught fish can improve the perception of wild-caught fish and change the opinion towards farmed fish [14,15]. However, we found some bias related to this topic. 

First, eco-labels are not fully understood: the authors of [16] demonstrated that the positive effect is significant only when additional information is provided as an explanation of the eco-label. Secondly, as stated above, on the one hand, the consumers’ interest in sustainable seafood is growing; on the other hand, it is controversial whether the presence of an eco-label can produce a positive effect in terms of real willingness to buy [15,17,18,19]. Indeed, some authors have shown how the price increase given by sustainable practices increases the final purchase price, often resulting in a barrier to consumption for consumers, especially if the sustainability criterion is not clear to them [20,21]. Thirdly, very few studies have analysed eco-labels' effect on real consumption. Most studies have observed the effect of the eco-labels on hypothetic liking by using questionnaires and simply showing related images [16,22].

In a study dating back to 2015, Simoes and colleagues [23] observed that the presence of a message about sustainability on packaging produced a higher freshwater prawn acceptability compared to a blind evaluation. However, they only provided a supplementary card with a sustainability message and did not use any specific eco-label. Moreover, they first tested the message effect on a single species of seafood, the freshwater prawn, so there might be a sample effect that is not demonstrable. Secondly, the freshwater prawns are farmed and not caught; therefore, the sustainability concept is limited. Nevertheless, Simoes and colleagues also reported that although sensory attributes are of primary importance when purchasing a food product, consumers who are not emotionally attached to particular products may be driven in their purchases by extrinsic and ethical factors. These factors generate consumer expectations of the product. Generally, consumers’ expectations of products depend on the characteristics of the product, but also on the characteristics of the consumer and the context in which the consumer and the product are located [24]. This means that product characteristics are perceived differently from consumer to consumer but also by the same consumer placed in different contexts [25]. Indeed, as [26] reported, a fruit beverage is perceived differently in different contexts by the same consumer (at breakfast, for refreshment, or while watching a movie). The expectation may be confirmed or disconfirmed during consumption [27,28].

In particular, confirmation occurs if the generated expectation reflects the sensory characteristics of the product, whereas disconfirmation occurs if the generated expectation does not reflect the sensory characteristics of the product [29,30]. The expectations generated can be of two types: sensory or hedonic. In the first case, the consumer believes the product possesses peculiar sensory characteristics with specific intensities, which may influence liking during consumption. In the second case, the consumer is influenced (positively or negatively) by information related to the product (sustainability, traceability, etc.) [30,31].

Therefore, according to what was stated above and the gaps observed in the literature, this study aimed to assess the impact of additional external information on fish liking evaluated by a group of Italian consumers. Specifically, the provided information consisted of a sustainability label on both raw and cooked European anchovy (*Engraulis encrasicolus*) and Bluefin tuna (*Thunnus thynnus*). The study was conducted by using a blind—expected—informed consumer test. The two species were chosen because both are threatened by overfishing (anchovies are considered a “poor fish”, while Bluefin tuna is a “fine fish”). The cause of overfishing for anchovies is that they are a very popular species, especially in southern Italy, where they are consumed in different food preparations. As they are gregarious species, they tend to be fished in large numbers, which damages the ecosystem; in addition, overcrowding in nets also causes tissue damage to some specimens, making them unsalable for the market. Bluefin tuna, on the other hand, is in great demand in both the Italian and Asian markets for its intense flavour and its use when raw; in fact, it is widely used in the preparation of sushi and sashimi but is also used in other preparations in cooked dishes. Therefore, efforts are being made to protect them so that stocks are not depleted by fishing pressures [32,33].

This study took part in the SUREFISH project, whose main objective was to valorise traditional Mediterranean fish by fostering supply-chain innovation and consumer confidence in Mediterranean fish products by deploying innovative solutions to achieve unequivocal traceability and confirming their authenticity, thus preventing fraud.

## 2. Materials and Methods

### 2.1. Consumer Sample

A convenient sample of 105 consumers (73 females and 32 males, average age 35 years), regular fish eaters, were involved in the consumer test. Consumers were recruited by word of mouth among people living in the Portici (Naples) area. Consumers were then contacted by email to book a date for the sessions, asking them to fill in a Doodle (Google content, Mountain View, CA, USA). With that email, consumers were also informed about data protection (DL 30.06.03, n. 196 and Reg. EU 2016/679) and their voluntary involvement.

### 2.2. Sample Preparation

The samples of anchovy (*Engraulis encrasicolus*, whole and slaughtered) and slices of tuna (*Thunnus thynnus*) were purchased from a local seller (Portici, Naples, Italy). Considering the characteristics of the samples (slaughtered and sliced), the ethical ap-proval was not required because the samples were not managed as alive. The test took 21 days to avoid great variability between species, and purchases were always made from the same seller. Specifically, the anchovies were purchased whole, while the tuna was purchased in slices. For the evaluation of the raw samples, the anchovies were beheaded, gutted, and washed with tap water immediately after purchase. The anchovies considered for evaluation had an average size of 15 ± 2 cm in length; smaller or larger samples were removed to have homogeneity among the evaluated samples and not to bias the evaluation. Regarding tuna samples, the skin and fat parts were removed, and then the slices were cut into 5 × 2 × 2 cm homogeneous cubes. For the evaluation of the cooked samples, the anchovies were floured with type 00 flour and sprayed with olive oil (to simulate a typical culinary preparation of southern Italy), while the tuna did not undergo any further treatment. Both samples were baked in a dry air oven at 270 °C for 5 min until reaching an internal temperature of 60–65 °C. The baking process was optimised during a preliminary experiment to prevent the sample from being too dry. The samples were freshly cooked; while the consumers evaluated the raw samples, the cooked samples were prepared and served freshly from the oven, with a serving temperature close to the cooking temperature.

### 2.3. Consumer Evaluation

The consumer test was carried out at the Sensory Laboratory of the Department of Agricultural Sciences (University of Naples Federico II) in Portici (Naples) under three experimental conditions—blind, expected, and informed—and applied in two different sessions (Session I: blind-expected conditions; Session II: informed condition) with two weeks of interval between each other. The average length of each session was about 15–20 min. All the sessions were carried out in individual booths, and the data were recorded using the Fizz 2.4 computerized system (Biosystèmes, Couterno, France). For all the experiments, the rules listed in the Declaration of Helsinki and the rules of the “Personal Data Protection Code”, Reg. EU 2016/679 of the European Parliament and of the Council of 27 April 2016 were applied as the primary ethical framework guiding our research. Specifically, all the food products used in this study are approved and regulated for human consumption. They do not represent new chemicals, substances, biomasses, or pharmaceuticals that would require specific ethical review. All the suppliers of the products used are certified producers for food sales in compliance with current regulations. Informed consent was obtained from all subjects involved in the study, ensuring the respect of privacy, participants’ rights and any information related to allergies or intolerances of participants to the products of our research.

Details of the two sessions are reported below.

- Session I

During the first session, consumers evaluated both anchovy and tuna samples without any information about them (blind condition). Raw and cooked samples were served to consumers in disposable plates coded with a three-digit numerical code. Raw samples were always assessed before cooked samples. The serving of the samples was randomised, so some consumers assessed the anchovy sample first and others the tuna sample. The consumers evaluated first the overall liking, then the appearance and the odour for the raw samples, and the overall liking, appearance, odour, flavour, and texture for the cooked samples.

The evaluation was conducted using the nine-point hedonic scale with the anchors “extremely disliked” (1) and “extremely liked” (9) for all the attributes. At the end of the blind evaluation, consumers first filled in a short questionnaire related to fish consumption. In particular, the first question was about the type of purchased fish, specifically the purchase frequency of fresh, frozen, canned, or transformed fish. The answers to this question were collected using a seven-point frequency scale with anchors 1 = “Never”, 4 = “Occasionally”, and 7 = “Always” [34]. The second question was about familiarity with the sustainability label Marine Stewardship Council (MSC). This question was assessed through the use of a five-point familiarity scale anchored with 1 = “I do not know it”, 2 = “I know it, but I never bought it”, 3 = “I bought it once”, 4 = “I bought it occasionally”, and 5 = “I usually bought it” [35]. Finally, the sociodemographic characteristics of consumers were collected. Specifically, age, gender, origin, education level, and annual income status were asked.

Secondly, consumers evaluated their expected liking for anchovy and tuna caught following the Marine Stewardship Council (MSC) sustainability label (expected condition) using the same scale used in the blind session.

- Session II

After two weeks, the same consumers were invited to attend the second and last session (informed condition). Thus, the informed evaluation followed the same course as the blind session, but samples were always served together with the MSC eco-label, and a written explanation of the latest was always available to be read. Therefore, consumers were informed that both fish species under test were caught according to the terms of the MSC sustainability label.

### 2.4. Statistical Analysis

Analysis of variance (one-way ANOVA, Duncan test *p* ≤ 0.05) was used to test the effect of sustainability information on overall liking scores collected in the different sessions (blind, expected, and informed). A paired-sample *t*-test was used to test how expectations generated by the sustainability label affected consumers’ liking scores. Specifically, expected liking minus blind liking (E-B), informed liking minus blind liking (I-B), and informed liking minus expected liking (I-E) were calculated. By using the *t*-test, it was established whether there were statistically significant differences. Repeated measures analysis of variance (Duncan test *p* ≤ 0.05) was used to test the correlation between consumer sociodemographic variables and blind, expected, and informed liking scores. Finally, a paired-sample *t*-test was used to verify any differences between the blind and informed scores for the two analysed fish species in both raw and cooked status. The analyses were conducted by using SPSS statistical software (SPSS vers. 29 Inc., Chicago, IL, USA).

## 3. Results and Discussion

### 3.1. Consumers’ Characteristics

Table 1 shows the sociodemographic characteristics of the consumers involved in the test. The age of the consumers was between 18 and 65 years (average 35 years old); for convenience, they were grouped into three age groups: 18–29, 30–44, and >45, according to [36,37]. Regarding their origin, the consumers came from both coastal and inland areas of the Campania region. Finally, regarding their education level, they had a middle school diploma, high school diploma, bachelor’s degree, and a master’s or Ph.D. degree.

### 3.2. Consumer Test

As reported in Table 2, one-way ANOVA (Duncan test *p* ≤ 0.05) analysis showed statistically significant differences in overall liking scores for both anchovy and tuna samples in the different test conditions (blind, expected, and informed) (*p* ≤ 0.05). In particular, the lowest score was given to the blind sample, whereas the samples with the sustainability label information (both expected and informed) were rated with higher liking scores.

The results are in agreement with other studies on different food matrices, including sheep vs. pork coppa [27], insect vs. vegetable vs. meat burger [38], or different brands of yoghurt (premium vs. commercial) [39], showing an improvement in overall liking when moving from the blind sample to samples with information (both expected and informed). Negative disconfirmation occurs in both anchovy and tuna samples (Table 2), as the expected minus blind liking was greater than 0. A paired-sample *t*-test showed statistically significant differences (*p* ≤ 0.05) between the blind and the expected (E-B) scores [39], thus showing high expectations for the species accompanied by the sustainability label information. The product external information also had an effect, as the informed liking minus the blind liking was also greater than 0, and the paired sample *t*-test showed statistically significant differences between those scores (I-B) (*p* ≤ 0.05). Thus, the informed samples were perceived better by consumers than the blind samples. By studying the ratio (I-B)/(E-B), as reported by [39], it is possible to determine whether the assimilation or contrast effects occurred. In particular, if that ratio is greater than 0, there is an assimilation effect; conversely, a contrast effect occurs if the ratio is less than 0. In this study, an assimilation effect occurs for both anchovy and tuna samples, as the above ratio showed results higher than 0, but this assimilation effect is incomplete as the value of informed liking minus expected (I-E) was less than zero and the paired-samples *t*-test showed statistically significant differences (*p* ≤ 0.05). This effect indicates that consumers’ expectations were not fully fulfilled by the information provided to them [40]. This means that expectations of liking for both sustainability-labelled fish species were very high compared to the liking assessed by consumers during the informed session. This discrepancy could be due to the lack of knowledge of the differences between sustainably and normally fished species without labels.

**Figure 1:**
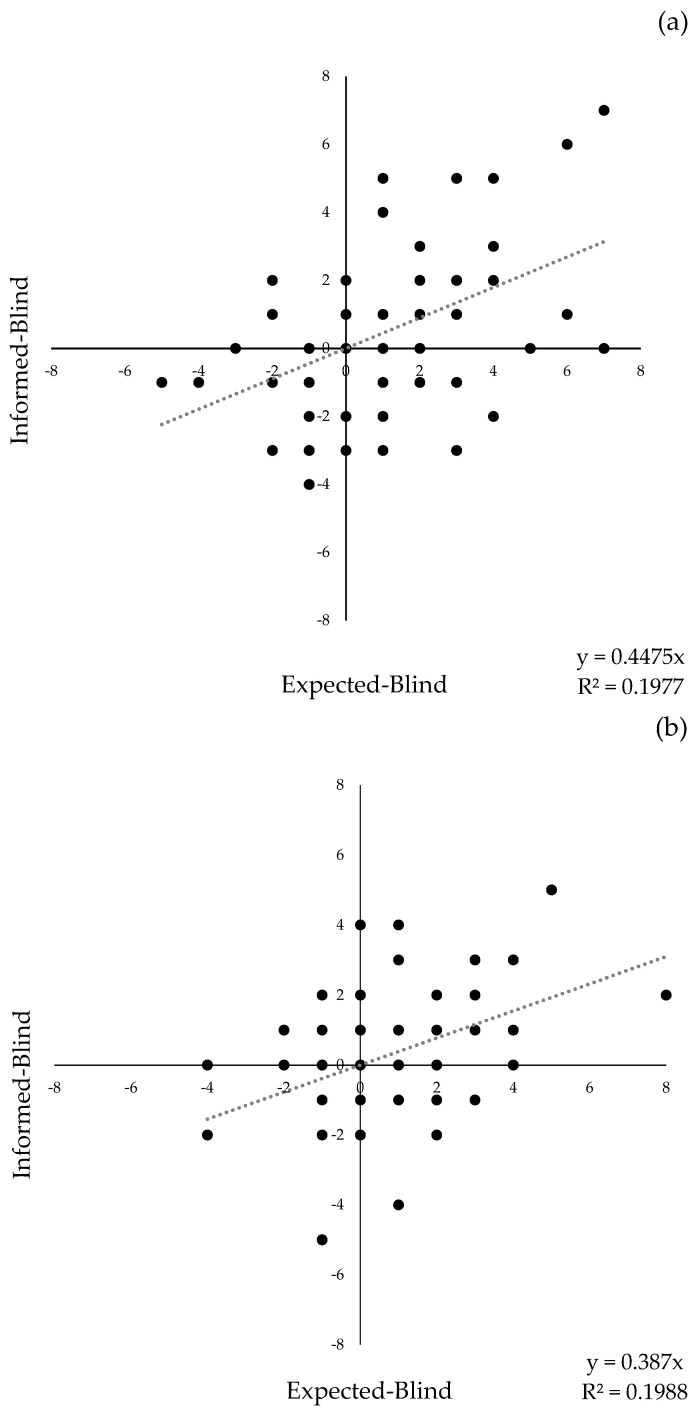
Plots of the effect of expectation disconfirm for Anchovies (**a**) and Tuna (**b**).

Figure 1 shows the graphs of the effect of information on the liking score (I-B) for each consumer as a function of the degree of disconfirmation (E-B) for both anchovies (Figure 1a) and tuna (Figure 1b). The effect of assimilation can also be seen in the graphs, as the linear regression shows a positive slope.

Thus, as confirmed above, during the informed session, the information about the sustainability label and the sensory characteristics of the tested samples caused the liking scores for both anchovies and tuna to be in the middle between the blind and the expected session. Although the consumers involved in the test were uninformed about sustainability, this topic interested them, so when external information was provided, they tended to magnify the product even without tasting it (expected liking) [38]. Indeed, as reported by several authors [41,42,43,44], emotional and ethical factors play a fundamental role during the purchasing phases, conditioning the consumers’ choices. For this same reason, the discrepancy between the samples in the informed and expected sessions could also be explained. Our results are in agreement with [45], which show how the expectations generated by a product, dependent on the information on the label, logos, shape, and colour of the packaging, can positively or negatively influence the consumer.

The short consumer questionnaire highlighted that the consumers’ purchasing choices were mainly oriented towards the purchase of fresh (mean score of 5.7 on a 7-point frequency scale), compared to frozen (mean score of 4.6), canned (mean score of 5.2), and processed (mean score of 3.4) fish products. This result may be due to the provenance from coastal areas of many consumers involved in the test. Indeed, as reported in other studies, coastal areas record higher consumption of fresh fish products due to their location close to the sea, which allows for daily supplies of fresh seafood products [46,47]. Regarding the familiarity with the MSC sustainability label, it turned out that the label was unfamiliar to consumers with an average score of 2.7 (score between the answer “I know it, but I have never bought it” and “I bought it once”). The unfamiliarity with the sustainability label may be due to the purchasing habits of the consumers involved, as they tend to buy more fresh fish, which always comes with less information on the label when compared to processed fish [3]. In addition, unfamiliarity may also be due to a lack of knowledge of the label and its meaning, thus making it go unnoticed during purchase [2]. Therefore, although consumers do not know the meaning of eco-labels for their consumption habits, they are interested in the topic of fish sustainability; this could further explain the high liking scores recorded in the expected session and, therefore, the incomplete assimilation effect obtained.

Table 3 shows the expected, blind, and informed liking values correlated with the sociodemographic variables of the consumers involved in the test. As can be seen in the table, a repeated-measures analysis of variance (Duncan test *p* ≤ 0.05) reported no statistically significant differences between the liking scores and the different correlated variables, except for age. In fact, subjects included in the 30–44 age group showed higher informed liking scores than the 18–29 and >45 group (*p* ≤ 0.05).

**Figure 2:**
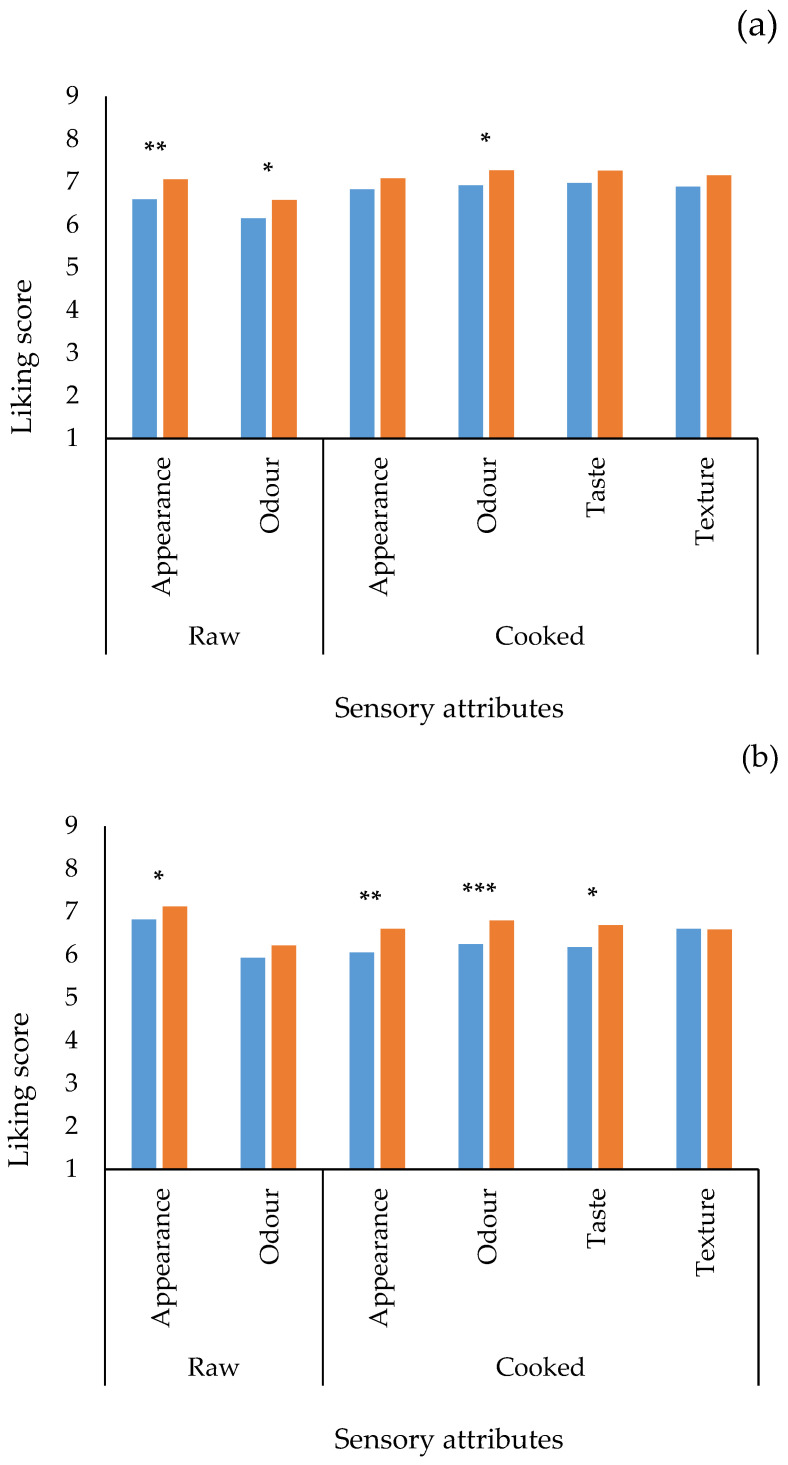
Average liking scores for the attributes assessed during the consumer test on a nine-point Likert-type hedonic scale (105 consumers). (**a**) Anchovies; (**b**) tuna. ■ Blind and ■ Informed. Asterisks indicate significant differences (* *p* < 0.05; ** *p* < 0.01; *** *p* < 0.001).

This result is in agreement with the study conducted by [3], who reported a greater involvement in sustainability on the part of medium-young Italian consumers (up to 44 years of age). The same study highlights that young people are less inclined to consume fish due to a series of barriers to consumption mainly related to the price, the taste and difficulty of preparing fish products in general. Therefore, by consuming less fish, younger consumers are also less aware of issues related to the world of fish [2]. Figure 2 shows the other hedonic attributes evaluated during the test for anchovies (Figure 2a) and tuna (Figure 2b).

As shown in the figure, all hedonic scores increased when the sustainability label information was provided to consumers. For anchovies, the appearance and odour attributes of the raw samples showed statistically significant differences between the blind and informed sessions. When cooked, only the odour showed statistically significant differences. Regarding tuna, the scores also increased with the sustainability information, except for the texture, which did not change if the sample was accompanied by the label. The appearance of the raw sample showed statistically significant differences between the two tests, while the differences were for the attributes of appearance, odour, and taste of the cooked samples. The results obtained are in agreement with other authors who emphasise the preference and the positive attitude of consumers towards buying sustainable fish products [48,49]. In general, as reported in other studies (not only focused on fish products) when the products to be evaluated are accompanied by more information, consumers’ final judgements will always tend to value the products accompanied by information compared to those evaluated in blind [40]. However, in the present study, the major effect of sustainability information is related to the tuna evaluation. This could be due to the fact that anchovies are perceived as a “poor” fish; therefore, overall, the liking is not so much influenced by additional quality information [50]. In the case of tuna, instead, its perception as a “fine” fish and the additional quality information synergistically work on consumers’ liking. Therefore, consumers seem to be less demanding for cheap species than for more expensive ones.

## 4. Conclusions

In conclusion, this study showed how consumers are influenced by extrinsic information related to a food product. The consumers involved in the study, probably due to a lack of knowledge about fish sustainability, tended to improve their hedonic perceptions of the evaluated species by moving from the blind to the informed session. The highest scores were recorded during the expected session, thus demonstrating a higher expectation of liking sustainable products than when the products were evaluated in the informed session. Consumers were positively oriented towards sustainable seafood products, although they were not fully aware of the MSC sustainability label, so their perception was altered by this information. Dissemination strategies aimed at improving knowledge about sustainable fish would, therefore, be necessary to orientate consumers’ purchasing choices towards these products. In particular, consumers should be informed about the quantities of fish that are landed each year to meet market demands, how many fish are landed, and which companies truly operate in a sustainable way. In this way, awareness of this topic can increase, and fisheries that are committed to respecting the environment can be valorised. Furthermore, to increase awareness among consumers and reduce the gap between the eco-label and its meaning, certification institutions, such as MSC, could be suggested to add QR codes that direct consumers to websites that explain in a simple way the meaning of the eco-label and the reduced impact that purchase has on the environment.

## 5. Limitations of the Study

The results should be viewed under the limitations of this study. The study was somewhat limited by the sample size (*n* = 105) and recruitment procedures employed. The genders of the study participants were not balanced (females = 73), and the age range needs to be expanded. The consumers involved were recruited in the same (limited) geographic area without considering variation across different socioeconomic segments, countries, and ethnicities.

## Figures and Tables

**Table 1:** Sociodemographic characteristics of the consumers involved in the study.

	Consumers (*n*)	Male (*n*)	Female (*n*)
Age			
18–29	49	14	35
30–44	30	8	22
>45	26	14	12
Provenance			
Seaside	65	22	43
Internal	40	14	26
Education			
Middle school diploma	6	3	3
High school diploma	30	15	15
Bachelor’s	52	13	39
Master’s/Ph.D.	17	5	12

**Table 2:** Average overall liking scores for anchovies and tuna during the blind (B), expected (E) and informed (I) sessions on a nine-point Likert-type hedonic scale (105 consumers).

Sample	E	B	I	E-B	I-B	I-E
Anchovy	7.3 ± 1.5 ^c^	6.3 ± 1.9 ^a^	6.8 ± 1.7 ^b^	0.98 ***	0.50 **	−0.5 *
Tuna	7.5 ± 1.5 ^b^	6.6 ± 1.5 ^a^	7.0 ± 1.4 ^a^	0.84 ***	0.36 *	−0.5 **

For each row, different letters correspond to statistically significant differences (Duncan test, *p* ≤ 0.05). Asterisks indicate significant differences (* *p* < 0.05; ** *p* < 0.01; *** *p* < 0.001).

**Table 3:** Correlation with sociodemographic variables.

		Gender			Age				Provenance		
Sample	Liking	M	F	*p*-Value	18–29	30–44	>45	*p*-Value	Seaside	Internal	*p*-Value
Anchovy	E	7.3	7.3	0.883	7.1	7.4	7.7	0.52	7.4	7.2	0.448
	B	6.2	6.4	0.689	5.9	7.0	6.4	0.200	6.4	6.1	0.462
	I	6.6	6.9	0.327	6.3 ^a^	7.4 ^b^	7.1 ^ab^	0.010	6.2	6.9	0.680
Tuna	E	7.5	7.5	0.948	7.2	7.8	7.7	0.111	7.4	7.5	0.423
	B	6.3	6.8	0.176	6.4	7.1	6.6	0.177	6.5	6.8	0.726
	I	6.9	7.0	0.722	6.8	7.2	7.2	0.319	6.9	7.1	0.596

For each row, different letters correspond to statistically significant differences (Duncan test, *p* ≤ 0.05).

## Data Availability

The original contributions presented in the study are included in the article, further inquiries can be directed to the corresponding author.

## References

[1] Ahmed N., Turchini G.M. (2021). The evolution of the blue-green revolution of rice-fish cultivation for sustainable food production. Sustain. Sci..

[2] García-Herrero L., De Menna F., Vittuari M. (2019). Sustainability concerns and practices in the chocolate life cycle: Integrating consumers’ perceptions and experts’ knowledge. Sustain. Prod. Consum..

[3] Fiorile G., Puleo S., Colonna F., Mincione S., Masi P., Solana N.H. (2023). Consumers’ Awareness of Fish Traceability and Sustainability: An Exploratory Study in Italy and Spain. Sustainability.

[4] van Bussel L.M., Kuijsten A., Mars M., van’t Veer P. (2022). Consumers’ perceptions on food-related sustainability: A systematic review. J. Clean. Prod..

[5] Weitzman J., Bailey M. (2018). Perceptions of aquaculture ecolabels: A multi-stakeholder approach in Nova Scotia, Canada. Mar. Policy.

[6] Stolzenbach S., Bredie W.L.P., Christensen R.H.B., Byrne D.V. (2013). Impact of product information and repeated exposure on consumer liking, sensory perception and concept associations of local apple juice. Food Res. Int..

[7] Nakaishi T., Chapman A. (2024). Eco-labels as a communication and policy tool: A comprehensive review of academic literature and global label initiatives. Renew. Sustain. Energy Rev..

[8] Boopendranath M.R. (2013). Seafood Ecolabelling. Fish. Technol..

[9] Maesano G., Di Vita G., Chinnici G., Pappalardo G., D’amico M. (2020). The role of credence attributes in consumer choices of sustainable fish products: A review. Sustainability.

[10] Carlucci D., Nocella G., De Devitiis B., Viscecchia R., Bimbo F., Nardone G. (2015). Consumer purchasing behaviour towards fish and seafood products. Patterns and insights from a sample of international studies. Appetite.

[11] Giacomarra M., Crescimanno M., Vrontis D., Miret Pastor L., Galati A. (2021). The ability of fish ecolabels to promote a change in the sustainability awareness. Mar. Policy.

[12] Miller A.M.M., Bush S.R. (2015). Authority without credibility? Competition and conflict between ecolabels in tuna fisheries. J. Clean. Prod..

[13] Blomquist J., Bartolino V., Waldo S. (2015). Price Premiums for Providing Eco-labelled Seafood: Evidence from MSC-certified Cod in Sweden. J. Agric. Econ..

[14] Bronnmann J., Hoffmann J. (2018). Consumer preferences for farmed and ecolabeled turbot: A North German perspective. Aquac. Econ. Manag..

[15] Uchida H., Onozaka Y., Morita T., Managi S. (2014). Demand for ecolabeled seafood in the Japanese market: A conjoint analysis of the impact of information and interaction with other labels. Food Policy.

[16] Donato C., D’Aniello A. (2022). Tell me more and make me feel proud: The role of eco-labels and informational cues on consumers’ food perceptions. Br. Food J..

[17] Mauracher C., Tempesta T., Vecchiato D. (2013). Consumer preferences regarding the introduction of new organic products. The case of the Mediterranean sea bass (*Dicentrarchus labrax*) in Italy. Appetite.

[18] Salladarré F., Brécard D., Lucas S., Ollivier P. (2016). Are French consumers ready to pay a premium for eco-labeled seafood products? A contingent valuation estimation with heterogeneous anchoring. Agric. Econ..

[19] Roheim C.A., Asche F., Santos J.I. (2011). The elusive price premium for ecolabelled products: Evidence from seafood in the UK market. J. Agric. Econ..

[20] Annunziata A., Mariani A., Vecchio R. (2019). Effectiveness of sustainability labels in guiding food choices: Analysis of visibility and understanding among young adults. Sustain. Prod. Consum..

[21] Aprile M.C., Punzo G. (2022). How environmental sustainability labels affect food choices: Assessing consumer preferences in southern Italy. J. Clean. Prod..

[22] Galati A., Miret-Pastor L., Siggia D., Crescimanno M., Fiore M. (2022). Determinants affecting consumers’ attention to fish eco-labels in purchase decisions: A cross-country study. Br. Food J..

[23] Simoes J.S., Mársico E.T., da Cruz A.G., de Freitas M.Q., Doro L.H., Conte-Junior C.A. (2015). Effect of sustainability information on consumers’ liking of freshwater prawn (*Macrobrachium rosenbergii*). J. Sci. Food Agric..

[24] de Andrade J.C., de Aguiar Sobral L., Ares G., Deliza R. (2016). Understanding consumers’ perception of lamb meat using free word association. Meat Sci..

[25] Zandstra E.H., Lion R. (2019). In-home testing. Context Eff. Environ. Prod. Des. Eval..

[26] Hein K.A., Hamid N., Jaeger S.R., Delahunty C.M. (2012). Effects of evoked consumption contexts on hedonic ratings: A case study with two fruit beverages. Food Qual. Prefer..

[27] de Andrade J.C., Nalério E.S., Giongo C., de Barcellos M.D., Ares G., Deliza R. (2018). Consumer sensory and hedonic perception of sheep meat coppa under blind and informed conditions. Meat Sci..

[28] Deliza R. (1995). External Cues and Its Effect on Sensory Perception and Hedonic Ratings: A Review. J. Sens. Stud..

[29] Murray J.M., Delahunty C.M. (2000). Mapping consumer preference for the sensory and packaging attributes of Cheddar cheese. Food Qual Prefer..

[30] Di Monaco R., Cavella S., Di Marzo S., Masi P. (2004). The effect of expectations generated by brand name on the acceptability of dried semolina pasta. Food Qual Prefer..

[31] Caporale G., Monteleone E. (2001). Effect of expectations induced by information on origin and its guarantee on the acceptability of a traditional food: Olive oil. Sci. Aliment..

[32] Ramírez F., Pennino M.G., Albo-Puigserver M., Steenbeek J., Bellido J.M., Coll M. (2021). SOS small pelagics: A safe operating space for small pelagic fish in the western Mediterranean Sea. Sci. Total. Environ..

[33] Jelić Mrčelić G., Nerlović V., Slišković M., Zubak Čižmek I. (2023). An Overview of Atlantic Bluefin Tuna Farming Sustainability in the Mediterranean with Special Regards to the Republic of Croatia. Sustainability.

[34] Saidi A., Sacchi G., Cavallo C., Cicia G., Di Monaco R., Puleo S., Del Giudice T. (2022). Drivers of fish choice: An exploratory analysis in Mediterranean countries. Agric. Food Econ..

[35] Tuorila H., Lähteenmäki L., Pohjalainen L., Lotti L. (2001). Food neophobia among the Finns and related responses to familiar and unfamiliar foods. Food Qual. Prefer..

[36] Predieri S., Sinesio F., Monteleone E., Spinelli S., Cianciabella M., Daniele G.M., Dinnella C., Gasperi F., Endrizzi I., Torri L. (2020). Gender, age, geographical area, food neophobia and their relationships with the adherence to the mediterranean diet: New insights from a large population cross-sectional study. Nutrients.

[37] Spinelli S., Prescott J., Pierguidi L., Dinnella C., Arena E., Braghieri A., Di Monaco R., Gallina Toschi T., Endrizzi I., Proserpio C. (2021). Phenol-rich food acceptability: The influence of variations in sweetness optima and sensory-liking patterns. Nutrients.

[38] Schouteten J.J., De Steur H., De Pelsmaeker S., Lagast S., Juvinal J.G., De Bourdeaudhuij I., Verbeke W., Gellynck X. (2016). Emotional and sensory profiling of insect-, plant- and meat-based burgers under blind, expected and informed conditions. Food Qual. Prefer..

[39] Schouteten J.J., De Steur H., Sas B., De Bourdeaudhuij I., Gellynck X. (2017). The effect of the research setting on the emotional and sensory profiling under blind, expected, and informed conditions: A study on premium and private label yogurt products. J. Dairy Sci..

[40] Grasso S., Rondoni A., Bari R., Smith R., Mansilla N. (2022). Effect of information on consumers’ sensory evaluation of beef, plant-based and hybrid beef burgers. Food Qual. Prefer..

[41] Desmet P.M.A., Schifferstein H.N.J. (2008). Sources of positive and negative emotions in food experience. Appetite.

[42] Paakki M., Kantola M., Junkkari T., Arjanne L., Luomala H., Hopia A. (2022). “Unhealthy = Tasty”: How Does It Affect Consumers’ (Un)Healthy Food Expectations?. Foods.

[43] Spinelli S., Masi C., Dinnella C., Zoboli G.P., Monteleone E. (2014). How does it make you feel? A new approach to measuring emotions in food product experience. Food Qual. Prefer..

[44] Thomson D.M.H., Crocker C., Marketo C.G. (2010). Linking sensory characteristics to emotions: An example using dark chocolate. Food Qual. Prefer..

[45] Piqueras-Fiszman B., Spence C. (2015). Sensory expectations based on product-extrinsic food cues: An interdisciplinary review of the empirical evidence and theoretical accounts. Food Qual. Prefer..

[46] EUMOFA (2020). The EU Fish Market 2020.

[47] Welch A., Lund E., Amiano P., Dorronsoro M., Brustad M., Kumle M., Rodriguez M., Lasheras C., Janzon L., Jansson J. (2002). Variability of fish consumption within the 10 European countries participating in the European Investigation into Cancer and Nutrition (EPIC) study. Public Health Nutr..

[48] Bronnmann J., Asche F. (2017). Sustainable Seafood From Aquaculture and Wild Fisheries: Insights From a Discrete Choice Experiment in Germany. Ecol. Econ..

[49] Groening C., Sarkis J., Zhu Q. (2018). Green marketing consumer-level theory review: A compendium of applied theories and further research directions. J. Clean. Prod..

[50] Gaviglio A., Demartini E., Mauracher C., Pirani A. (2014). Consumer perception of different species and presentation forms of fish: An empirical analysis in Italy. Food Qual. Prefer..

